# Supernatant Metabolites from Halophilic Archaea to Reduce Tumorigenesis in Prostate Cancer* In-vitro *and* In-vivo*

**Published:** 2019

**Authors:** Atefeh Safarpour, Marzieh Ebrahimi, Seyed Abolhassan Shahzadeh Fazeli, Mohammad Ali Amoozegar

**Affiliations:** a *Department of Stem Cells and Developmental Biology, Cell Science Research Center, Royan Institute for Stem Cell Biology and Technology, ACECR, Tehran, Iran. *; b *Department of Developmental Biology, Faculty of Basic Sciences and Advanced Technologies in Biology, University of Science and Culture, ACECR, Tehran, Iran.*; c *Department of Molecular and Cellular Biology, Faculty of Basic Sciences and Advanced Technologies in Biology, University of Science and Culture, ACECR, Tehran, Iran. *; d *Microorganisms Bank, Iranian Biological Resource Centre (IBRC), ACECR, Tehran, Iran.*; e *Extremophiles Laboratory, Department of Microbiology, School of Biology and Center of Excellence in Phylogeny of Living Organisms, College of Science, University of Tehran, Tehran, Iran.*

**Keywords:** Prostate cancer, Supernatant metabolites, Nude mice, Archaea, Halophile

## Abstract

Halophilic archaea are known as the novel producers of natural products and their supernatant metabolites could have cytotoxic effects on cancer cells. In the present study, we screened the anticancer potential of supernatant metabolites from eight native haloarchaeal strains obtained from a culture collection in Iran. Five human cancer cell lines including breast, lung, prostate and also human fibroblast cells as the normal control were used in the present study. Moreover, to evaluate the anti-tumor effect of the selected supernatant, inhibition of sphere formation and tumor development was assessed *in-vitro* and *in-vivo*, respectively. Among all strains, supernatant metabolites from *Halobacterium salinarum* IBRC M10715 had the most potent cytotoxic effect on prostate cancer cell lines (IC50 = 0.5 mg/mL) without any effects on normal cells. It significantly increased both early and late apoptosis (about 11% and 9%, respectively) in the androgen-dependent PC3 cell line, reduced sphere formation ability of DU145 and PC3 cells with down-regulation of *SOX2* gene expression. Furthermore, our results revealed that tumors developed in nude mice significantly shrank post intratumor injection of metabolites of the haloarchaeal strain. In conclusion, we suggested here for the first time that supernatant metabolites from *Halobacterium salinarum* IBRC M10715 could be a novel component against prostate cancer *in-vitro* and *in-vivo* with remarkable reduction in stem-like properties of tumor.

## Introduction

Considered as a lethal disease, cancer causes millions of death every year. Despite the new molecular and targeted therapies, several cancers return after various treatments (1). Cancer stem cells are subpopulations in cancer, which cause drug resistance and tumor recurrence. Screening for new products with the ability to kill these cells is a novel approach to cancer therapy (2). Among many sources of obtaining natural products, microorganisms are known as the fast and easy producers of bioactive molecules. Their short lifespan makes them a good candidate for finding new bioactive molecules with anticancer activity (3). Up to now several natural products with anticancer activity have been found. Among them, salinomycin from *Streptomyces albus* has been reported with potent activity for inhibiting and killing cancer stem cells and currently, it’s in phase III of clinical trials (4-6).

Extremophiles are considered as a proper source for maximizing the available chemical diversity from microorganisms. Living in extreme conditions enables them to produce interesting bioactive compounds with novel therapeutic activities (7). Some metabolites from extremophile microorganisms with cytotoxic activity against breast cancer cell line (MCF-7) have been introduced (8).

Halophilic microorganisms are the extremophiles with the ability to live in high salt concentrations. Living in these harsh conditions enables them to produce unique metabolites with various effects, which may not find in the non-extreme part of life. Thus, halophiles can be a good source of bioactive compounds or secondary metabolite with novel activities (9, 10). In this regards, 8-O-Methyltetrangulol and Naphthomycin A derived from *Streptomyces* sp. nov. WH26; as a halophilic bacterium; have been introduced to have cytotoxic activity against A549, HeLa, BEL-7402, and HT-29 (11). Furthermore, carotenoids from halophile archaeal strains have exhibited cytotoxic activity against HepG2 cancer cells (12, 13). In spite of all above evidence, the investigations in this field are still inadequate, therefore studies to find novel components from halophilic microorganisms with anti-cancer effect are necessary. 

In the present study, eight native haloarchaeal strains obtained from Iranian biological resource center (IBRC) were studied. The cytotoxic effect of supernatant metabolites (SM) from all strains were screened on five different cancer cell lines including lung (A549), prostate (DU145, PC3), breast (MCF-7, MDA-MB-468) and human fibroblast cells as the normal control. The anti-tumoral activity of selected strain was assessed *in-vitro* and *in-vivo* using nude mice model. Based on our knowledge, for the first time in the present study, we report the anti-cancer effect of supernatant metabolites from halophilic archaea against prostate cancer.

## Experimental

*Chemicals and Cell lines*

All the materials and reagents for archaeal cultures were purchased from Merck (E. Merck, Darmstadt, Germany). Ethyl acetate for SM extraction was purchased from Samchun (Korea). 3-(4,5-Dimethylthiazol-2-yl)-2,5-diphenyl tetrazolium bromide (MTT), Dulbecco’s modified Eagle’s medium (DMEM) with high glucose, FBS (Fetal Bovine Serum), trypsin, penicillin, and streptomycin were purchased from Biosera (Austria). Ultra-purified water was used throughout the analysis and all other chemicals were analytical grade. 

Prostate cancer cell lines (PC3 and DU145) were obtained from national cell bank of Iran (Pasteur Institute of Iran, Tehran, Iran). Breast cancer cell lines of MDA-MB-468 (IBRC C10095) and MCF-7 (IBRC C10082) and lung cancer cell line (A549; IBRC C10080), were obtained from cell bank of Iranian Biological Resource Center, Tehran, Iran. Human foreskin fibroblast (HFF-5) cell line was obtained from Royan Institute stem cell bank.

*Archaea culture and supernatant isolation*

Eight strains from halophilic archaeal strains were selected. The archaeal strains were obtained from IBRC microorganisms bank (Table 1). All strains were cultured in Modified Growth Medium (MGM) with 23% (w/v) of total salt (14). For SM production, a loopful of agar slant culture of each halophilic archaeal strain was inoculated into 25 mL of MGM. The inoculated medium was incubated at 40 °C for 48 h on a rotary shaker at 150 rpm, and then transferred into 225 mL medium to cultivate at the same temperature for 7 days.

*Metabolite extraction from supernatant*

The archaeal culture medium was removed and centrifuged at 4000 g for 40 min. The cell-free supernatant was collected after centrifugation and was filtered with Whatman no.1 filter paper. The equal volume of ethyl acetate was added to the supernatant with the ratio of 1:1 and it was shaken well for 2 h at room temperature. Then, it was allowed to settle overnight in a stand, at 4 °C. The upper organic layer containing SM was collected into a clean and dry bottle and was evaporated by BUCHI Rotavapor R_114 (Switzerland) until 1 mL of total volume remained in the rotary balloon. The remaining volume was transferred to a sterile vial with an identified weight and later on, it was dried completely by rotary. The dried SM was weighted and the pure weight of the SM was calculated. Approximately 3.5 mg of SM were obtained from 1 L of each archaeal culture. The SM was dissolved in DMSO (Merck, Germany), serving as a stock solution, which was later diluted to a final solvent concentration. The SM was tested as the weight of total SM/volume (11).

*Human Cell culture*

Human prostate cancer cells (DU145, PC3), breast cancer cells (MDA-MB-468, MCF-7), lung carcinoma (A549) and human foreskin fibroblast cells (HFF-5 as normal control) were grown as a monolayer in DMEM supplemented with 10% (v/v) fetal bovine serum (FBS), 1.0 mM sodium pyruvate and 2 mM L-glutamine,100 U/mL penicillin and 100 μg/mL streptomycin at 37 °C in a humidified atmosphere of 95% air and 5% CO_2_. 

*MTT assay*

The MTT-assay was used to evaluate the effect of the SM on cell viability pre and post treatment. Briefly, 5000 cells/well were seeded onto a 96-well plate and were allowed to adhere for overnight. Cultivated cells were incubated with different concentrations of SM (0, 0.01, 0.1, 0.2, 0.4 and 0.8 mg/mL) for 48 h. The final concentration of DMSO was <1 mM that was not cytotoxic for the cells. To assess the viability of the cells, 10 μL of 5 mg/mL solution of MTT in PBS was added to each well and incubated for 3 h at 37 °C. Finally, the medium was removed and 100 μL of the DMSO was added to each well to solubilize the blue formazan. Dye absorbance was measured at 560 nm (13). One chamber of each 96-well plate contained only DMSO and its OD_560_ assumed as blank. All of the ODs (control and treated groups) from each plate first subtract from the blank of the same plate and then the mean of OD_560_ for control and treated groups were calculated. The percentage of viable cell calculate via mean OD_560 (Treated group)_/mean OD_560 (Control group)_ × 100. 

*Apoptosis test*

The apoptotic effects of SM on treated and control cells were measured using the AnnexinV/PI Staining Kit (Sigma) according to the manufacturer’s instruction. Binding of annexin-V to phosphatidylserine suggests early apoptosis, whereas binding to both annexin-V and PI indicates late apoptosis.

*Sphere formation assay*

Sphere formation capacities were assessed after 48 h of metabolite pretreatment. To do this first, the cells were seeded at a density of 10^6 ^cells/well in 6-well plate and allowed to adhere to plate for 24 h. Then they were treated by the SM IC50 dose for 48 h. Un-treated cells were used as control group. Then the cells were harvested and followed to sphere formation assay. Briefly, 500 cells/cm^2 ^were seeded in culture dishes coated with (2-hydroxyethyl methacrylate) (poly-HEMA, Sigma) and cultivated in serum free DMEM supplemented with B27 (GIBCO, Karlsruhe, Germany), 20 ng/mL EGF and bFGF (Royan Institute, Tehran, Iran) and PenStrep. The cells were grown for 7 days and maintained in a humidified incubator at 37 °C and an atmospheric pressure of 5% (v/v) carbon dioxide/air. Then, the spheres with diameters of >50 µm were counted using an eyepiece graticule. The percentage of plated cells which formed sphere was calculated, and was referred as the percentage of sphere formation. The values were expressed as means ± SD of at least three independent experiments.

*Quantitative real-time RT-PCR (qRT-PCR)*

Total mRNA was isolated from treated and control cell lines after 48 h using TRIzol Reagent (Invitrogen) according to the manufacturer’s instruction. cDNA was synthesized using the RevertAid H Minus First Strand cDNA Synthesis Kit (Fermentas, Waltham, Massachusetts, USA). SYBR Premix Ex Taq II (Tli RNase H Plus) (Takara, Berkeley, California, USA) was utilized to perform quantitative real-time reverse transcription-PCR (RT-PCR) using Rotor-Gene 6000 (Corbett). The *GAPDH* gene transcript was measured as a normalizer to determine the other gene relative transcripts (2^-ΔΔCt^). The sequences of primers are listed in Table 2.

*Mouse Tumorigenicity Assay*

The suspension of 3 × 10^6^ cells (PC3, DU145) was prepared in 20 µL of matrigel (0.34 mg/mL, BD Bioscinces, San Jose, CA, USA). Six Nude female mice in the ages of 4-6 weeks (were purchased from Pasteur Institute of Iran and maintained under clean area provided by HEPA-Filtered Laminar-Air-Flow Systems). The animals were allowed to acclimate for two weeks prior to experimentation. All mice were injected with the 3 × 10^6 ^cells subcutaneously, one injection on the flank in each animal (six mice with PC3 and six with DU145). Once tumors got palpable (about 3 days post injection), tumor volumes were calculated using the formula: (*L *× *W*^2^)/2 which L and W indicated length and width, respectively. The length and width were measured with a caliper every other day. To find the proper dose of SM intra-tumor injection we considered the 0.5 mg/mL dose of *in-vitro* injection as 500 mg/kg (15, 16). Because the minimum weight of our mice was 17.5 g, we injected 8.75 mg of SM intratumorally in each mouse in the treated group. We select the minimum dose to avoid the death of mice. The SM was dissolved in DMSO (total volume 70 µL) and then intra-tumor injection was done. The tumors of one PC3 and one DU145 injected mice were injected with only DMSO (70 µL) as the control. The mice were sacrificed in a humane manner when the tumor size exceeds 25 mm in diameter in either direction.

*Immunohistochemistry*

Isolated tumors were fixed by paraformaldehyde (4% V:V) and then horizontal serial sections at 4 mm thickness were prepared by using a Leica RM2125RT microtome (Leica RM2125RT, Leica Microsystems Inc., Bannockburn, IL). For immunohistochemically assays, the sections were deparaffinized, and subjected to antigen retrieval (antigen retrieval PH9, Dako) for 30 min. Sections were rinsed thoroughly with phosphate-buffered saline containing 0.05% tween-20 (PBST) and endogenous peroxidase activity was quenched with 1% H2O2 in methanol for 30 min in the dark. To increase the permeability, the sections were treated with Triton X-100 (0.5%) for 10 min and then blocked with 10% goat serum for one hour (17). Then, the slides were incubated with primary antibody human Ki67 antibody (1:100, Santa Cruz Biotechnology, INC., USA) at 4 °C, overnight; afterwards, the slides were washed with PBST and incubated for one hour at 37 °C with horseradish peroxidase (HRP) conjugated secondary antibodies (Abcam6789 1:500 dilution). Finally, color was developed by treating samples with 3, 3›-diaminobenzidine (DAB; Sc-2051, Santa Cruz) for 5 min. The sections were then washed with distilled water, counterstained with hematoxylin, dehydrated with two changes of alcohol, cleared by xylene and cover slipped. The labeled cells were visualized using Olympus BX51 microscope and images were captured with an Olympus DP70 digital camera under the same setting. Measurement of positive Ki67 positive cells was performed in ten randomly selected areas of five pictures from three different replicates with an eyepiece graticule. The means of the different experimental groups were compared by ANOVA. The differences were considered to be statistically significant at *p *< 0.05.

*Statistical analysis*

One-way ANOVA test was used to determine the statistical significance of the differences between the treated and the control groups. All of the experiments were performed in triplicates or more and the data obtained were expressed as means ± SD. *p *< 0.05 was considered as statistically significant.

## Results

*Screening of haloarchaeal strains with anti-cancer potential*

Eight haloarchaeal strains were obtained from IBRC microorganisms bank, Tehran, Iran. All of the strains were isolated from Aran-Bidgol hypersaline lake in the center of Iran. Seven of them were type strains while one of them (*Halobacterium* *salinarum*) was not (Table 1). 

The viability of human fibroblast cells (HFF-5; as normal cells), prostate cancer lines (DU145, PC3), breast cancer lines (MDA-MB-468, MCF-7) and lung cancer cell line (A549) was evaluated post-treatment with each supernatant metabolites (SM). As shown in Table 1, the SM of *Haloarchaeobius* *iranensis* was not selected because it had no significant effect on viability of cancerous cells (Figure S1) and only substantial reduced viability of PC3 and A549 in 0.8 mg/mL concentration. In contrast, *Halovenus* *aranensis, Halorientalis* *persicus, *and* Halopenitus malekzadehii *induced a significant reduction in viability of A549, MCF-7, and MDA-MB-468 in concentrations of 0.4, 0.8, and 0.8 mg/mL, respectively, but also reduced significantly the viability of HFF-5 cells in those concentrations (Table 1 and Figures S2, S3, and S4). Two species including *Halopenitus* *persicus* and *Halovivax* *limisalsi* decreased the viability of HFF-5 cell significantly at 0.01 mg/mL concentration but had no effect on cancer cell viability in this concentration (Table 1 and Figures S5 and S6). Therefore, we exclude them from the rest of the study. Although, *Halovivax* *cerinus *caused a significant decrease in the viability of DU145 cells, it was not selected for further studies because its effect was not including both cell lines of a cancer type (Table 1 and Figure S7). 

**Figure 1:**
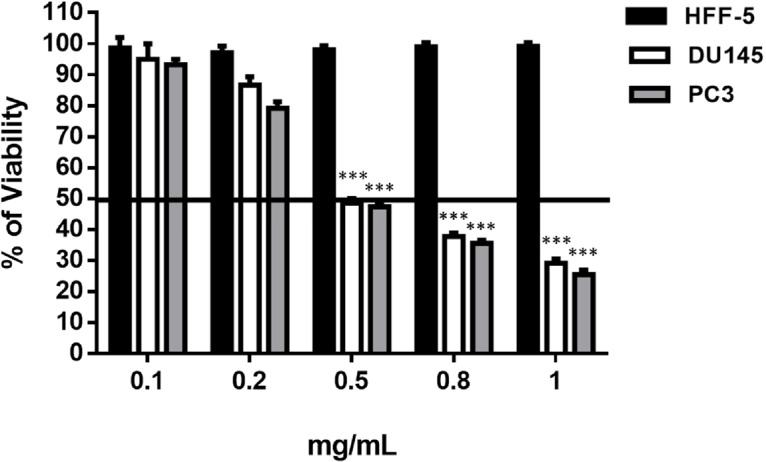
Dose determination of *Halobacterium salinarum *Supernatant Metabolite on prostate cancer cell lines (PC3 and DU145) and fibroblast cell line (HFF-5). SM at different concentrations was added to the culture medium of PC3, DU145 and HFF-5. The data represents mean ± SD. *p*-values showed significance of viability decrease. ***p < 0.001

Among all strains, *Halobacterium* *salinarum* strongly reduced cell viability of prostate cancer cell lines at the concentrations of 0.4, 0.5 and 0.8 mg/mL, without any adverse effect on HFF-5 cell line (Table 1, Figures 1 and S8). We observed a 50% reduction in cell viability in DU145 and PC3 48 h post-treatment (Figure 1). Hence, this strain was selected for the further experiments.

*Halobacterium salinarum’s supernatant metabolites induce late apoptosis in prostate cancer cells*

**Figure 2:**
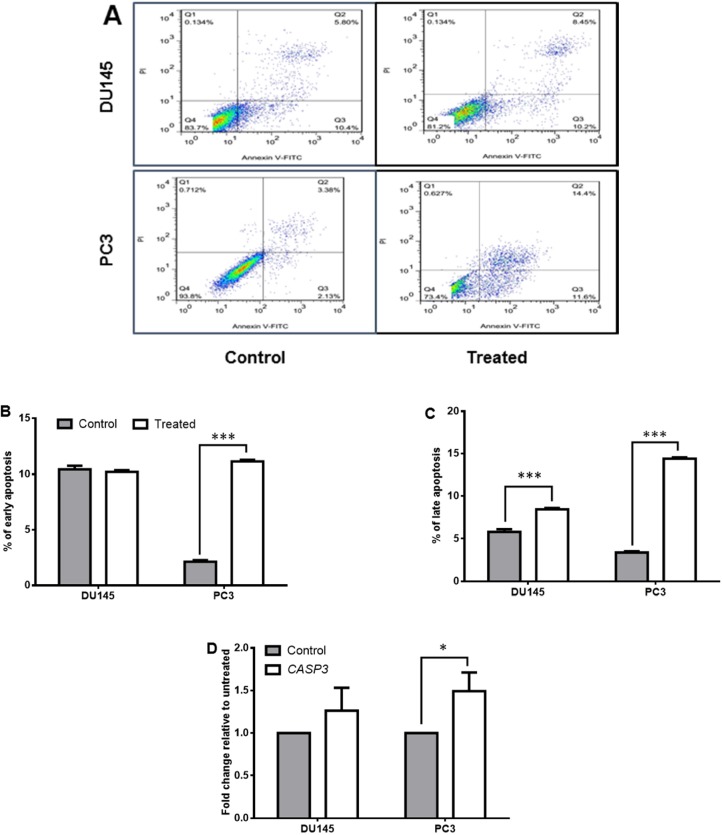
Effect of *Halobacterium salinarum *IBRC M10715 supernatant metabolite on apoptosis of prostate cancer cell lines (DU145 and PC3). (A) Apoptosis test of *Halobacterium salinarum *SM on DU145 and PC3 cells was done by annexin-PI staining, two days post treatment. Downright quadrate shows cells in early apoptosis and upright quadrate shows late apoptosis. (B) The comparison of early apoptosis and (C) late apoptosis in both DU145 and PC3 cells post 48 h treatment with 0.5 mg/mL of *Halobacterium salinarum *SM.

The Annexin/PI assay was done on treated DU145 and PC3 cells to seek the effect of *Halobacterium salinarum *IBRC M10715 SM on induction of apoptosis. As shown in Figures 2A-2C the percentage of both early and late apoptosis significantly enhanced in PC3 post-treatment with *Halobacterium salinarum *SM. However, its effect on DU145 was just on promoting the late apoptosis (Figures 2A-2C). The expression of *CASP3* (the gene encoding caspase3; a marker of late apoptosis) also significantly up regulated just in PC3 line (*p *< 0.05, Figure 2D).

*Prostate cancer stem cell properties are affected by Halobacterium salinarum IBRC M10715 supernatant metabolite in-vitro and in-vivo*

**Figure 3:**
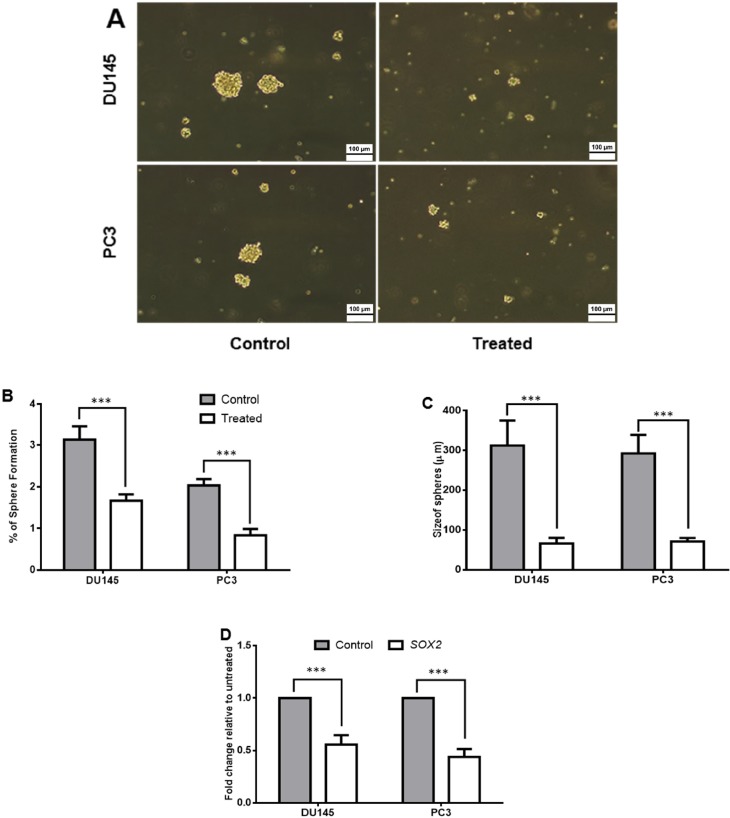
The effect of *Halobacterium salinarum *IBRC M10715 supernatant metabolite on stemness properties of prostate cancer cell lines. (A) 106 cells of treated with 0.5 mg/mL concentration of *Halobacterium salinarum *SM per T25 flask were subjected to sphere formation in no-adherent conditions. Untreated cells were used as control group. (B) The percentage of spheres pre and post treatment was calculated by dividing the number of spheroids to the number of seeding number of seeding number ´ 100. (C) Size of spheres in pre and post 48 h treatment of PC3 and DU145. (D) The relative expression of *SOX2 *gene (as pluripotency marker) was assessed by quantitative real-time PCR. The *GAPDH *was used for normalizing of data. Bars indicated Mean± SD of at least 3 biological replicates, (***p < 0.001).

Sphere formation *in-vitro* and tumor development *in-vivo* can determine self-renewal potential and tumorigenicity of cancer cells (18). Therefore, we evaluated the effect of SM of *Halobacterium* *salinarum* on sphere formation ability of DU145 and PC3 cells. Our results indicated that both cells in treated groups lost their repopulation capacity in which sphere formation was reduced from 3.1% and 2.0% to 1.6% and 0.8% in DU145 and PC3 cells, respectively (*p *< 0.001) and it was more dominant in PC3 cells (Figures 3A-3C). Moreover, the size of spheres was reduced in both treated cell lines (from 312.5 µm to 66.25 µm with *p *< 0.001 in DU145 and from 292.5 µm to 71.25 µm with *p *< 0.001 in PC3). To further confirm these results, quantitative real-time PCR was done for *SOX2*, as a pluripotency gene. *SOX2* expression down-regulated significantly in both treated cell lines (*p *< 0.001) and was more pronounced in PC3 (Figure 3D).

**Figure 4:**
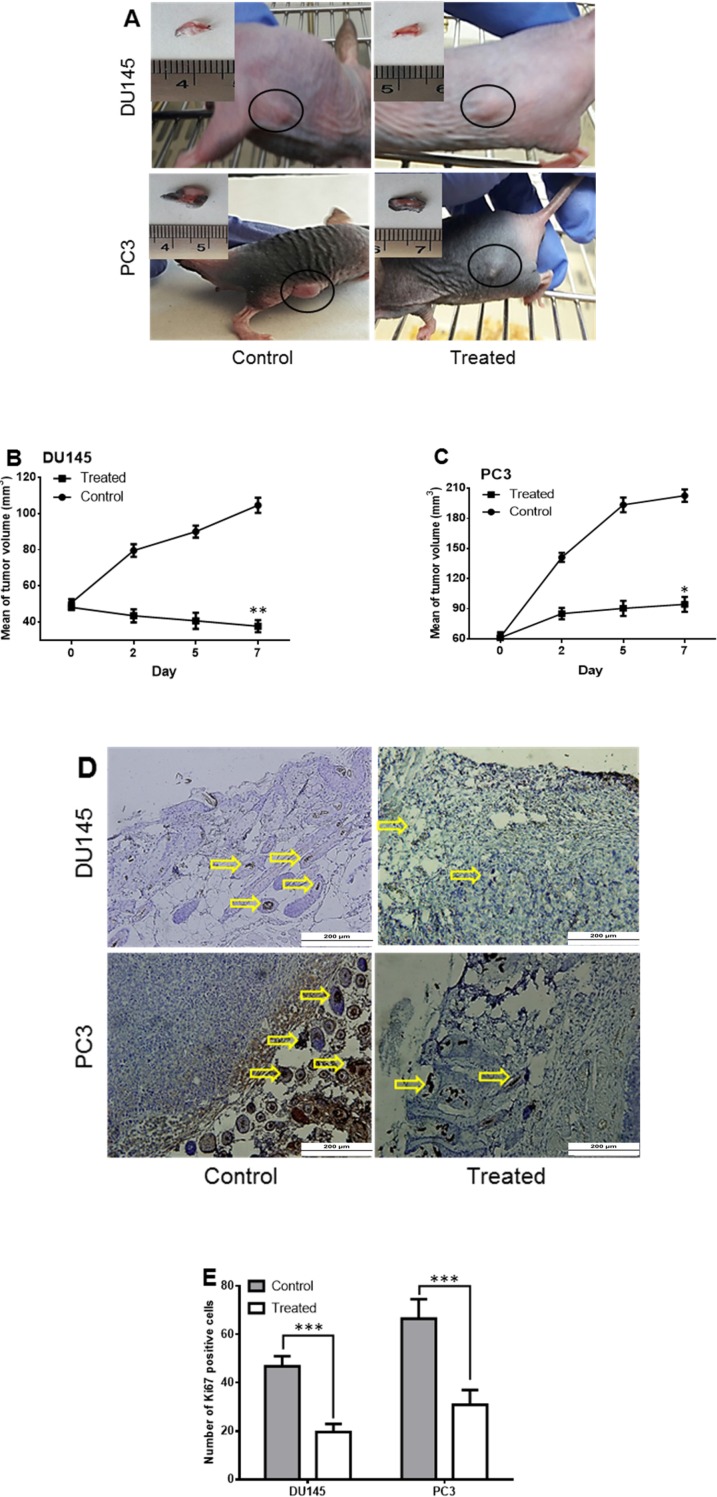
Tumorigenicity of *Halobacterium salinarum *IBRC M10715 SM treated prostate cancer cells in nude mice. (A) Tumors developed in nude mice 3 days post injection of 3 × 106 DU145 and PC3 cells per 20 μL of matrigel. The *Halobacterium salinarum *SM was injected intra tumoraly. In control group equal volume of DMSO injected intratumoraly. The tumor volume developed by (B) DU14 and (C) PC3 cells was calculated by measuring length and width for seven days. (D) Immunostaining of Ki67 in control and treated tumors. Yellow arrows indicate brown nuclei of positive cells. (E) The Ki67 positive cells was counted under the light microscope. Bars indicated mean ± SD of at least 10 randomly selected filed from five pictures in each group. *p < 0.05, **p < 0.01, ***p < 0.001

In the next step, the tumorigenicity of treated cells was checked using the subcutaneous injection of 1 × 10^6 ^of DU145 and PC3 cells to nude mice. When the size of tumor reached to 50-60 mm^3^ (about seven days), the SM of *Halobacterium salinarum* IBRC M10715 was injected intratumorally, just for one time. Interestingly, the sizes of tumors (Figures 4A-4C) were smaller compared to that of controls post seven days of SM injection (104.6 mm^3^ *vs.* 37.656 mm^3^ in DU145 and 202.7 mm^3^ *vs.* 94.4.16 mm^3^ in PC3), and this eradication was more prominent in the PC3 group. Immunostaining of human Ki67 (a marker of proliferation) using specific antibody indicated the intense reduction in Ki67 positive cells (yellow arrows) in treated mice (Figure 4D). 

The number of Ki67 positive cells decreased from 66.5 ± 8.07 and 46.8 ± 4.21 to 30.9 ± 6.10 and 19.6 ± 3.38 in PC3 and DU145 cells, respectively (Figure 4E).

## Discussion

In recent decades, cancer has been one of the most significant fatal factors, killing thousands every year. In spite of various methods used for treatment, they are still impotent to cure cancer in lowercase (19). Since 2007, after a decade of quiescence, investigations on utilizing natural compounds to treat cancer have been restarted. Among diverse resources, microorganisms have gained huge interest and attention as the more potent and valuable reservoir of natural products (3). Archaea are the group of microorganisms which has always been interesting to researchers due to their carotenoids (12, 20). Furthermore, the halophilic archaea due to their extreme living environments have attracted the attention of several scientists (13, 14). Living in such harsh conditions has given them the ability to produce peculiar and unique molecules and metabolites, whereas other organisms are unable to make similar ones (20).

**Table 1:** Effect of Supernatant Metabolite of selected archaeal strains on the viability of normal and cancer cells

IBRC No.	Species	HFF-5	PC3	DU145	A549	MDA- MB-468	MCF-7
			0.8 µg/mL	0.8 µg/mL			
IBRC M10715	*Halobacterium* *salinarum*	-	37.7 ± 1.0	35.5 ± 1.2	-	-	-
			*p < *0.001	*p < *0.001			
		0.8 µg/mL					0.8 µg/mL
IBRC M10043	*Halorientalis* *persicus*	51.0 ± 2.4	-	-	-	-	39.0 ± 4.1
		*p < *0.001					*p < *0.001
		0.8 µg/mL				0.8 µg/mL	
IBRC M10418	*Halopenitus* *malekzadehii*	62.2 ± 0.7	-	-	-	60.6 ± 0.4	-
		*p < *0.05				*p < *0.001	
			0.8 µg/mL		0.8 µg/mL		
IBRC M10013	*Haloarchaeobius* *iranensis*	-	62.2 ± 1.7	-	68.5 ± 1.8	-	-
			*p < *0.05		*p < *0.05		
		0.1 µg/mL					
IBRC M10041	*Halopenitus* *persicus*	70.6 ± 1.0	-	-	-	-	-
		*p < *0.05					
		0.4 µg/mL			0.4 µg/mL		0.8 µg/mL
IBRC M10015	*Halovenus* *aranensis*	67.8 ± 2.5	-	-	66.1 ± 1.5	-	60.8 ± 3.8
		*p < *0.05			*p < *0.05		*p < *0.01
				0.4 µg/mL			
IBRC M10256	*Halovivax* *cerinus*	-	-	60.9 ± 1.2	-	-	-
				*p < *0.05			
		0.01 µg/mL					
IBRC M10022	*Halovivax* *limisalsi*	70.5 ± 0.9	-	-	-	-	-
		*p < *0.05					

-: The SM did not reduce the viability of cells significantly in any of examined concentrations. HFF-5: Fibroblast cell line. PC3 and DU145: Prostate cancer cell lines.

A549: Lung cancer cell line. MDA-MB-468 and MCF-7: Breast cancer cell lines.

* Data shown as percentage of viability ± SD. *p*-values showed significance of decrease in viability.

**Table 2:** Primer sequences for Real-time PCR

**Gene**	**Forward primer**	**Reverse primer**
*GAPDH*	5' GTG GTC TCC TCT GAC TTC AAC 3'	5' AGG GTC TCT CTC TTC CTC TTG 3'
*SOX2*	5' CAA GAT GGC CCA GGA GAA C 3'	5' TCA TGT AGG TCT GCG AGC TG 3'
*CASP3*	5' AAG CGA ATC AAT GGA CTC TGG 3'	5' CAA GTT TCT GAA TGT TTC CCT GAG 3'

(D) Quantitative real-time PCR analysis of *CASP3*, as marker of late apoptosis, after 48 h treatment with 0.5 mg/mL of *Halobacterium salinarum *SM in PC3 and DU145. *p < 0.05, ***p < 0.001.

Recently, there have been studies on the anti-cancer effects of the carotenoids produced by halophilic archaea and induction of apoptosis in cancer cells by them (12, 13). However, no reports exist about the anticancer effects of metabolites produced by halophilic archaea. Thus, in the present study, we screened supernatant metabolites (SM) of at least eight strains of halophilic archaea isolated from a hypersaline lake in Iran. Based on our initial screening, the SM of *Halobacterium salinarum* reduced the viability of prostate cancer cell lines in lower concentrations while it was ineffective on normal cells (HFF-5). This suggested that their effects might be cancer cell type dependent, which have been previously reported by Sagar (21, 22) who observed the more potent effect of halophilic bacteria extracts on HeLa, MCF-7 and DU145 cells (21, 22). To find the mechanism of *Halobacterium salinarum*’s SM on prostate cancer cells, the rate of apoptosis was assessed. We found that the percentage of late apoptosis and the expression level of *CASP3* significantly were enhanced in both DU145 and PC3 cells. Although the present study is the first study which has evaluated the anti-cancer effect of supernatant metabolites from archaea, induction of apoptosis in cancer cells previously has been reported by archaeal carotenoids (12, 13). Also, induction of apoptosis by supernatant metabolites of four moderately halophilic bacteria in Human Umbilical Vein Endothelial Cells (HUVEC) and Mesenchymal Stem Cells (MSCs) has been reported previously (23). Moreover, a bioactive molecule (PPDHMP) from a marine bacterium *Staphylococcus* sp. strain MB30 induces apoptosis in the lung (A549) and cervical (HeLa) cancer cells through increasing the expression of *CASP3* (24). During the data analysis, we observed the increasing level of early apoptosis in treated PC3 cells as androgen-dependent prostate cancer cells. Since androgen-independent DU145 cell has been reported to be more sensitive to chemical drugs (17, 25 and 26), therefore derivatives and metabolites of *Halobacterium salinarum* could be effective in combination with other current therapies. Like any other cancer cell lines, sphere formation is a demonstrator of stem-like cells existing in prostate cancer lines (27). Lower amounts of spheroids and their smaller sizes mean that the stem-like ability which is responsible for tumor growth and recurrence has been reduced (28). Our results indicated that *Halobacterium salinarum* could significantly reduce the number and size of spheres. Moreover, the expression of *SOX2* as a pluripotency gene, was down-regulated in treated cells that confirm the results of sphere formation ability. As *SOX2* was assumed to be a significant marker to evaluate the progression of prostate cancer (29), the treatment with metabolites from *Halobacterium* *salinarum* IBRC M10715 could prevent the progression of human prostate cancer. Also, it was reported that the inhibition of *SOX2* in DU145 cells reduced proliferation and induced apoptosis in these cells (29). Therefore, it is probable that the increase in late apoptosis in these cells was correlated to *SOX2* downregulation.

*In-vivo* examinations showed that the tumor size decreased in the presence of SM and especially PC3 tumors displayed a higher decrease which was expected from *in-vitro* results. In line with our results, Halophilic bacterial extracts exhibited tumor growth inhibition ability in previous studies (30). It was reported that ethyl acetate extracts from the co-culture of *Streptomyces* sp. ANAM-5 and AIAH-10 had decreased tumor growth in Ehrlich Ascites Carcinoma cell bearing albino mice (30). Staining with Ki67 antibody has shown that proliferation of tumor cells was decreased in treated cells in comparison to the untreated ones. There is a strong positive correlation between high Ki67 expression and high-grade histopathology in neoplasms in the PC3 cell line (31). Therefore, the metabolites of* Halobacterium* *salinarum* could decrease neoplasms in treated cells by decreasing Ki67 positive cells.

## Conclusion

The results of the present study indicated the major anti-cancer effect of supernatant metabolites derived from *Halobacterium* *salinarum*. They can induce apoptosis by overexpression of *CASP3*, reduce pluripotency through downregulation of *SOX2* and cause the eradication of tumors in the mouse model. Moreover, we suggested *Halobacterium salinarum* SM as an anti-cancer agent which can greatly affect the androgen-dependent prostate cancers. 

This research is the first report on the anti-cancer effects of the archaeal supernatant metabolites. Additional studies and extensive screenings could result in introducing new compounds with stronger anti-cancer effects on prostate cancer. 
