# Supplementary material for: Supernatant Metabolites from Halophilic Archaea to Reduce Tumorigenesis in Prostate Cancer In-vitro and In-vivo
Source: Iran J Pharm Res. 2019 Winter;18(1):241–53. (PMC6487416)
Supplement: Supplementary file 1 — supplementary material [file ijpr-18-241-s001.pdf]

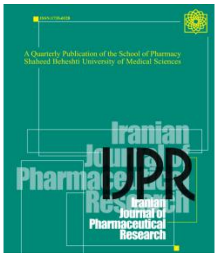

Supplementary Materials for

Supernatant Metabolites from Halophilic Archaea to Reduce Tumorigenesis in Prostate Cancer *In-vitro* and *In-vivo*

Atefeh Safarpour, Marzieh Ebrahimi*, Seyed Abolhassan Shahzadeh Fazeli* and Mohammad Ali Amoozegar

*To whom correspondence should be addressed. E-mail: Mebrahimi@royaninstitute.org; fazeli@ibrc.ir

Volume 18, Issue 1 (Winter 2019)

This PDF file includes:

Figures S1 to S8

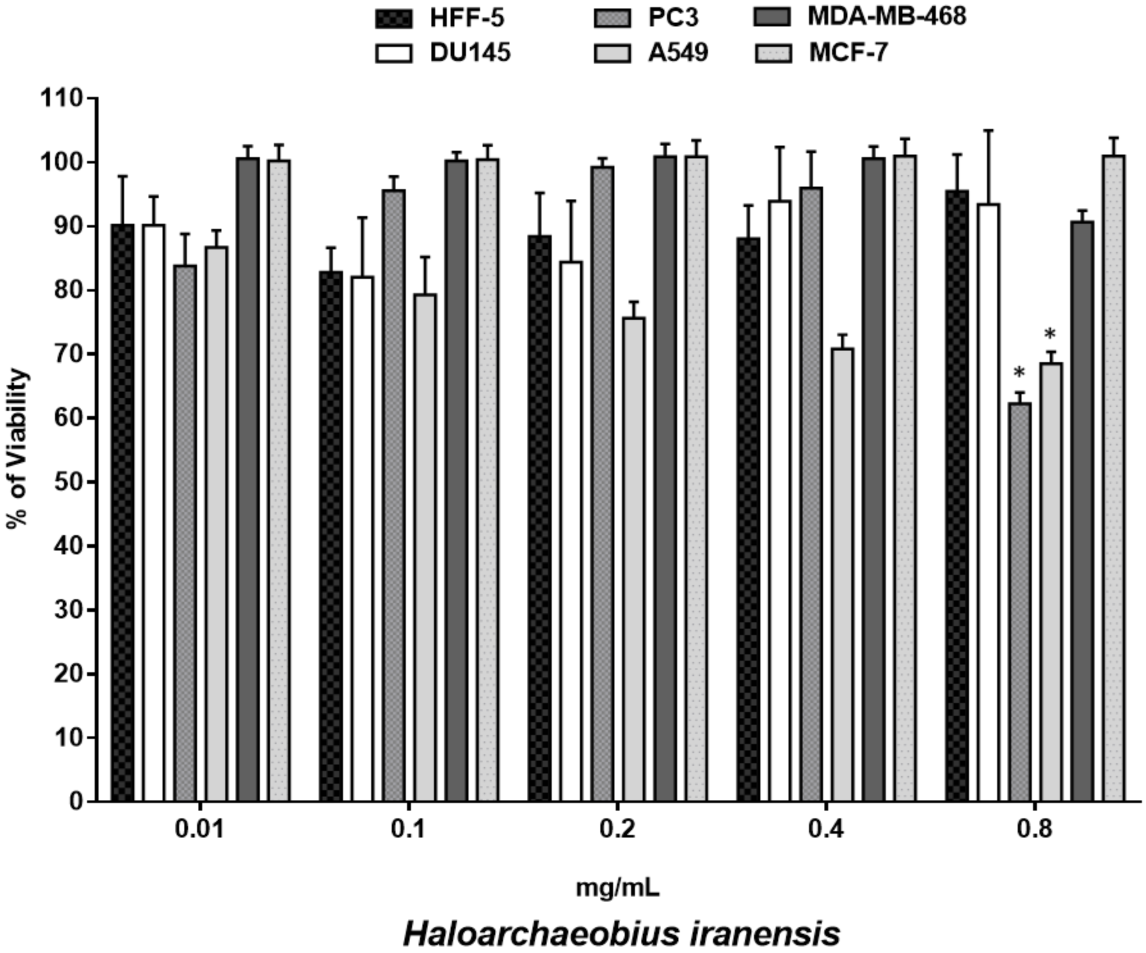

Figure S1. Screening of Supernatant Metabolites (SM) from *Haloarchaeobius iranensis* on viability of prostate (PC3 and DU145), breast (MCF7 and MDA-MB-231), lung (A549) cancer cell lines and human foreskin fibroblast (HFF-5). The viability of cells measured 48 h post treatment with supernatant metabolites from *Haloarchaeobius iranensis* by MTT. HFF-5 was used as normal control group. Bars indicated mean ± SD at least in five different biological replications. *p-values* showed significance of viability decrease. $^{*}p < 0.05$.

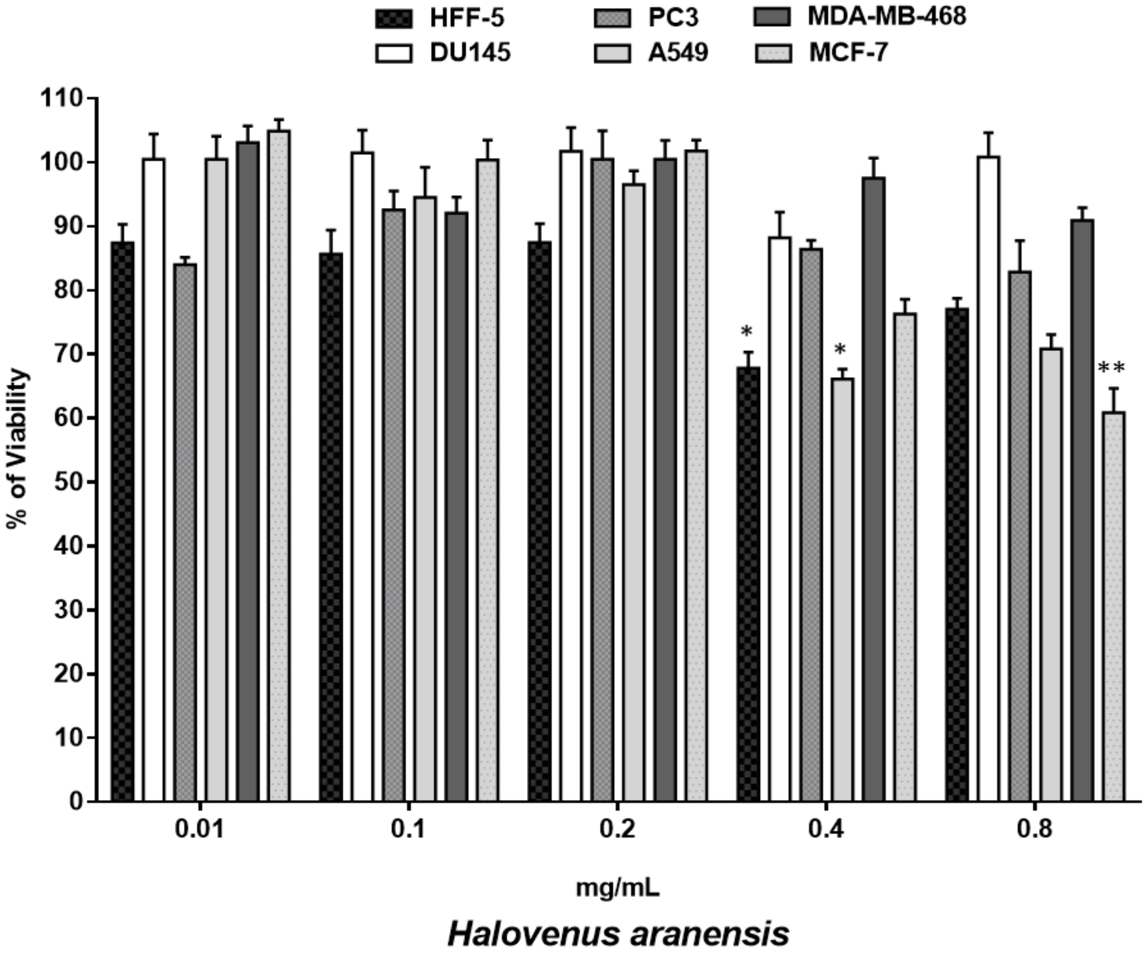

Figure S2. Screening of Supernatant Metabolites (SM) from *Halovenus aranensis* on viability of prostate (PC3 and DU145), breast (MCF7 and MDA-MB-231), lung (A549) cancer cell lines and human foreskin fibroblast (HFF-5). The viability of cells measured 48 h post treatment with supernatant metabolites from *Halovenus aranensis* by MTT. HFF-5 was used as normal control group. Bars indicated mean ± SD at least in five different biological replications. *p-values* showed significance of viability decrease. $^{*}p < 0.05$, $^{**}p < 0.01$.

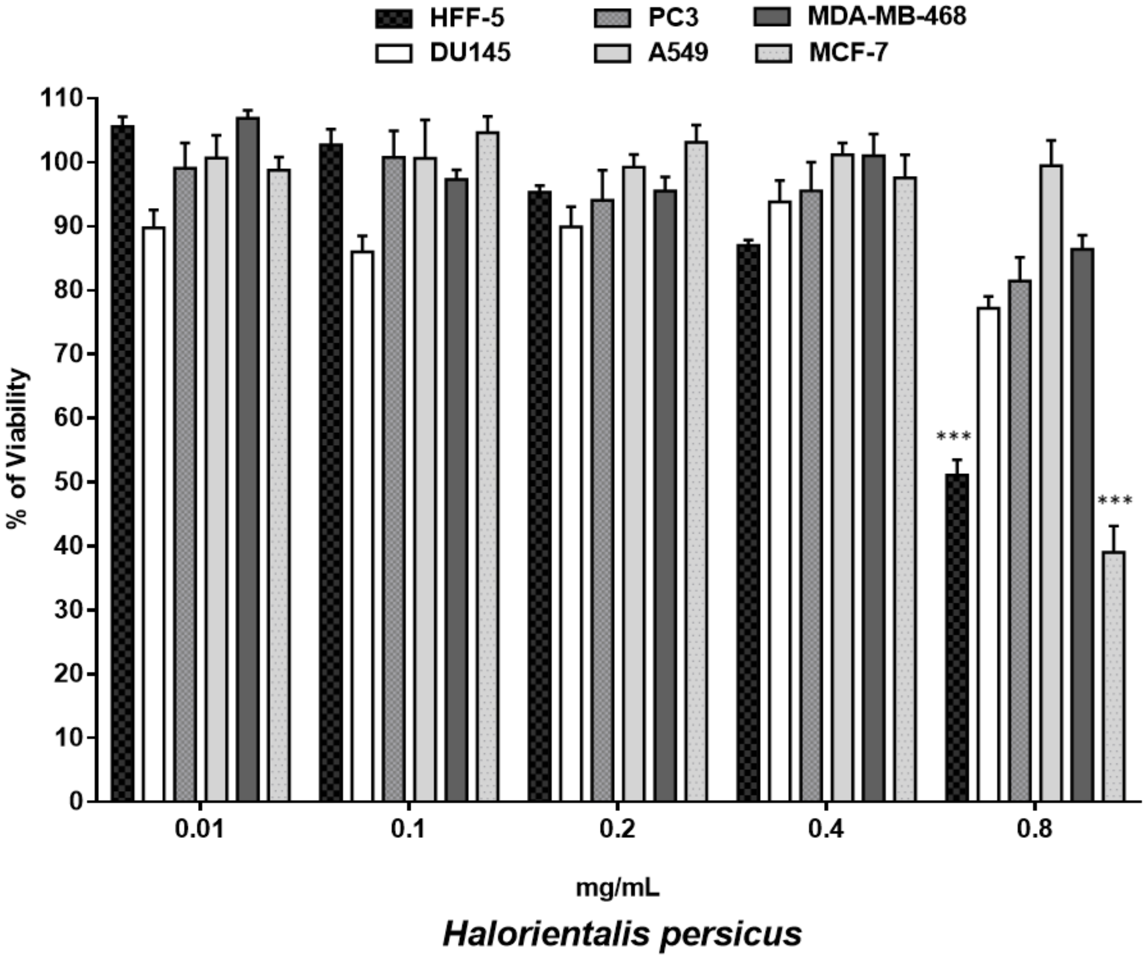

Figure S3. Screening of Supernatant Metabolites (SM) from *Halorientalis persicus* on viability of prostate (PC3 and DU145), breast (MCF7 and MDA-MB-231), lung (A549) cancer cell lines and human foreskin fibroblast (HFF-5). The viability of cells measured 48 h post treatment with supernatant metabolites from *Halorientalis persicus* by MTT. HFF-5 was used as normal control group. Bars indicated mean ± SD at least in five different biological replications. *p-values* showed significance of viability decrease. $^{***}p < 0.001$

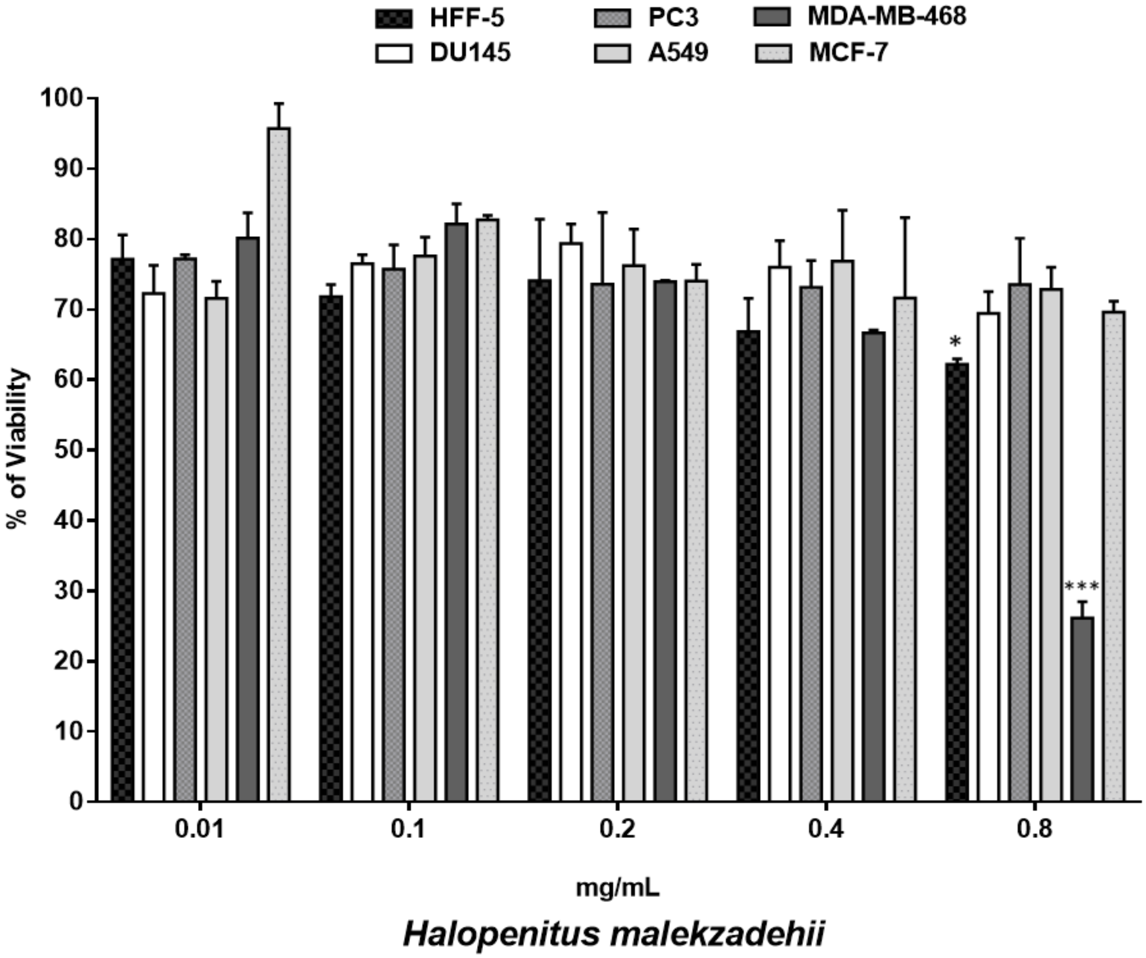

Figure S4. Screening of Supernatant Metabolites (SM) from *Halopenitus malekzadehii* on viability of prostate (PC3 and DU145), breast (MCF7 and MDA-MB-231), lung (A549) cancer cell lines and human foreskin fibroblast (HFF-5). The viability of cells measured 48 h post treatment with supernatant metabolites from *Halopenitus malekzadehii* by MTT. HFF-5 was used as normal control group. Bars indicated mean ± SD at least in five different biological replications. *p-values* showed significance of viability decrease. $^{*}p < 0.05$, $^{***}p < 0.001$.

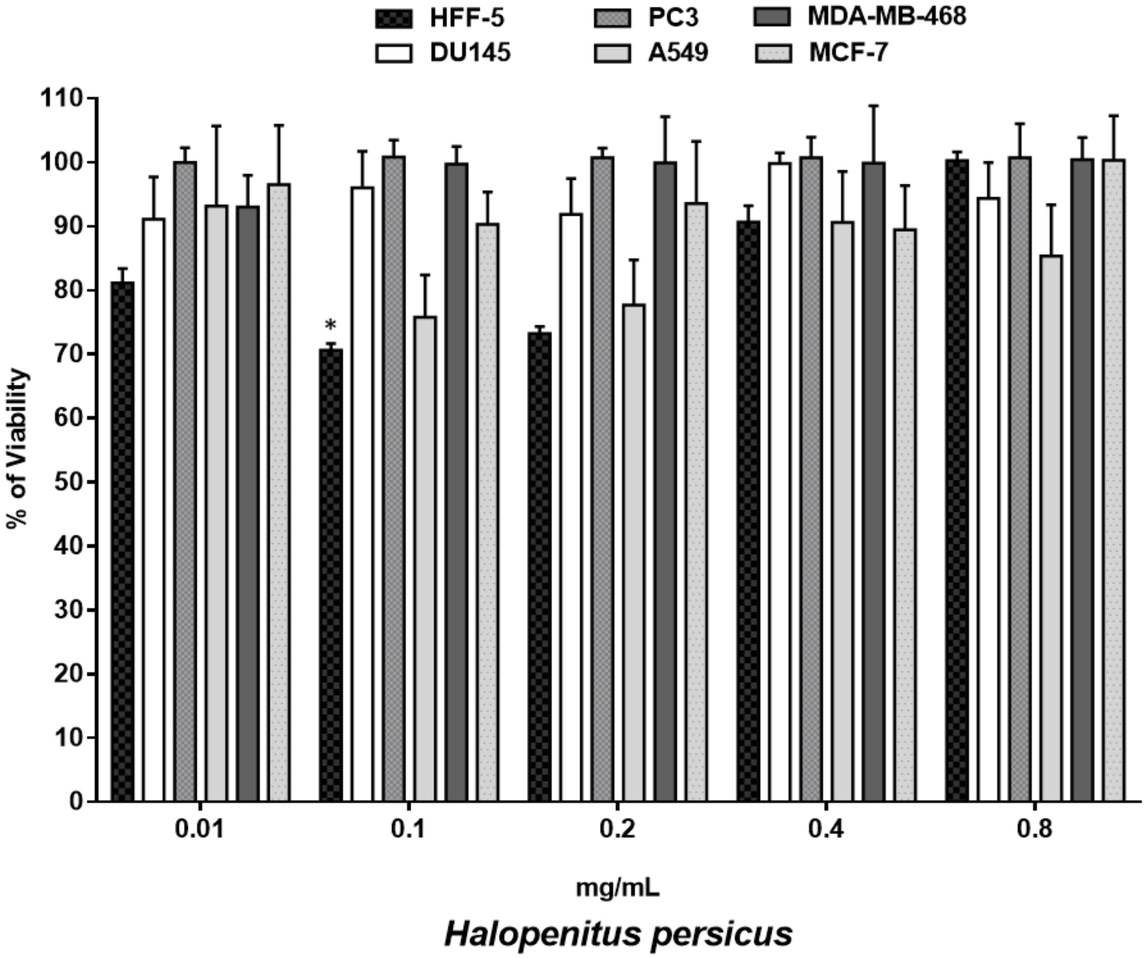

Figure S5. Screening of Supernatant Metabolites (SM) from *Halopenitus persicus* on viability of prostate (PC3 and DU145), breast (MCF7 and MDA-MB-231), lung (A549) cancer cell lines and human foreskin fibroblast (HFF-5). The viability of cells measured 48 h post treatment with supernatant metabolites from *Halopenitus persicus* by MTT. HFF-5 was used as normal control group. Bars indicated mean ± SD at least in five different biological replications. *p-values* showed significance of viability decrease. $^{*}p < 0.05$.

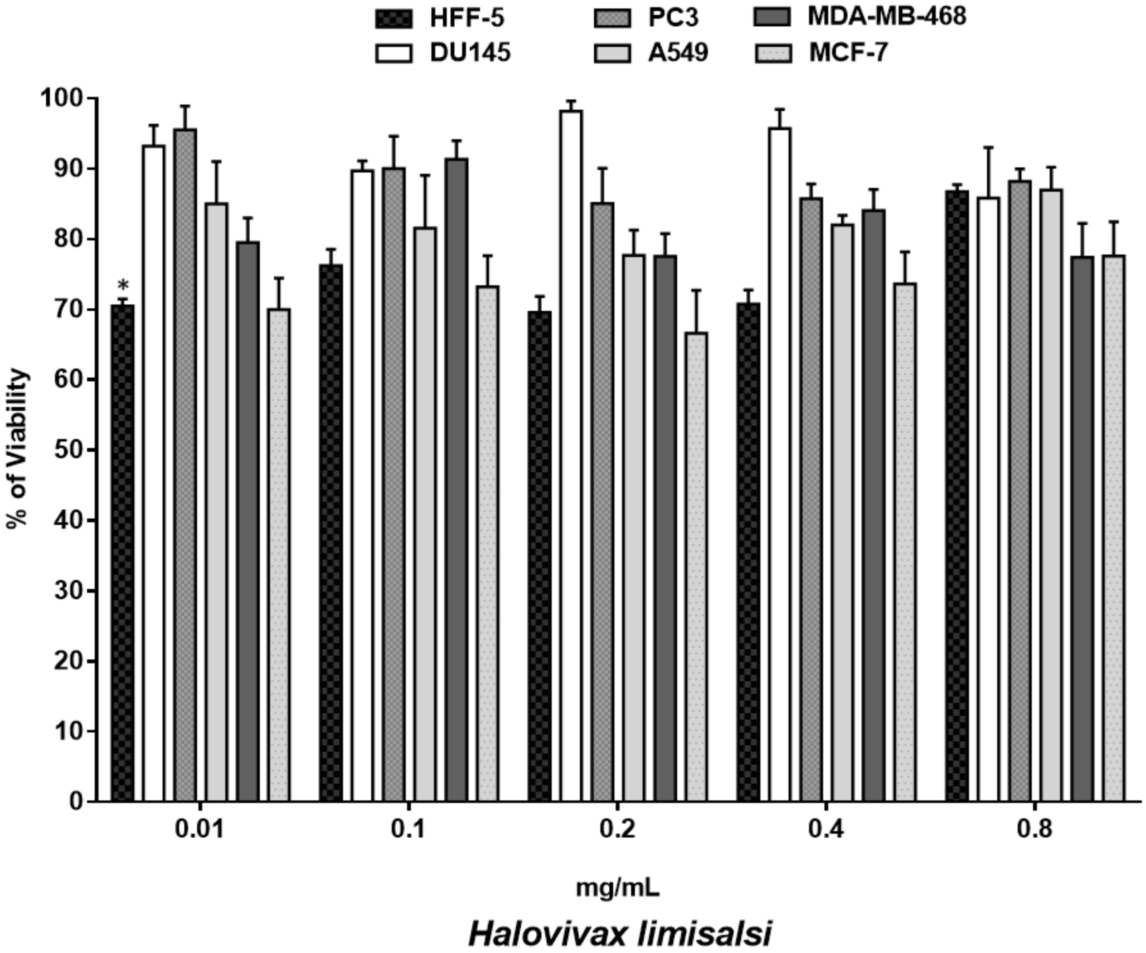

Figure S6. Screening of Supernatant Metabolites (SM) from *Halovivax limisalsi* on viability of prostate (PC3 and DU145), breast (MCF7 and MDA-MB-231), lung (A549) cancer cell lines and human foreskin fibroblast (HFF-5). The viability of cells measured 48 h post treatment with supernatant metabolites from *Halovivax limisalsi* by MTT. HFF-5 was used as normal control group. Bars indicated mean ± SD at least in five different biological replications. *p-values* showed significance of viability decrease. $^{*}p < 0.05$.

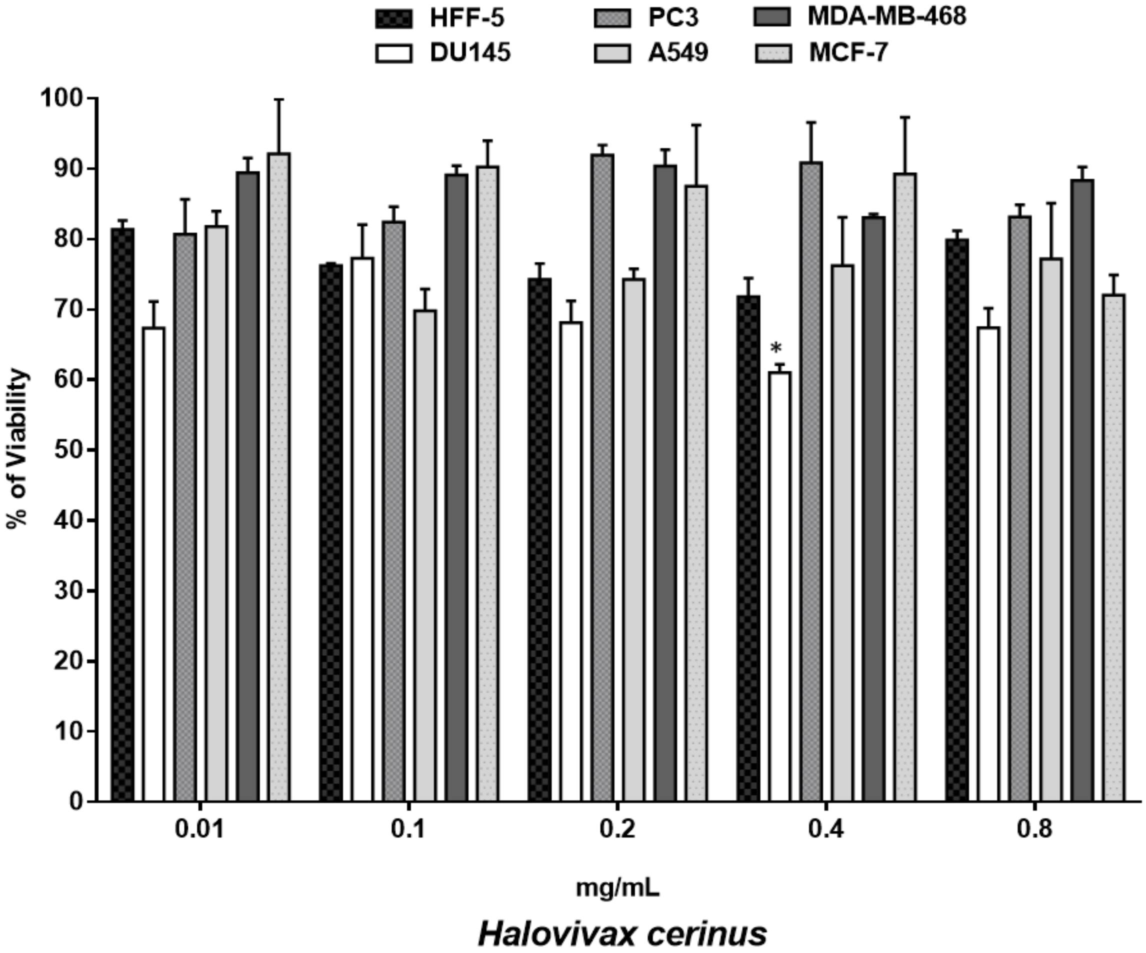

Figure S7. Screening of Supernatant Metabolites (SM) from *Halovivax cerinus* on viability of prostate (PC3 and DU145), breast (MCF7 and MDA-MB-231), lung (A549) cancer cell lines and human foreskin fibroblast (HFF-5). The viability of cells measured 48 h post treatment with supernatant metabolites from *Halovivax cerinus* by MTT. HFF-5 was used as normal control group. Bars indicated mean ± SD at least in five different biological replications. *p-values* showed significance of viability decrease. $^{*}p < 0.05$.

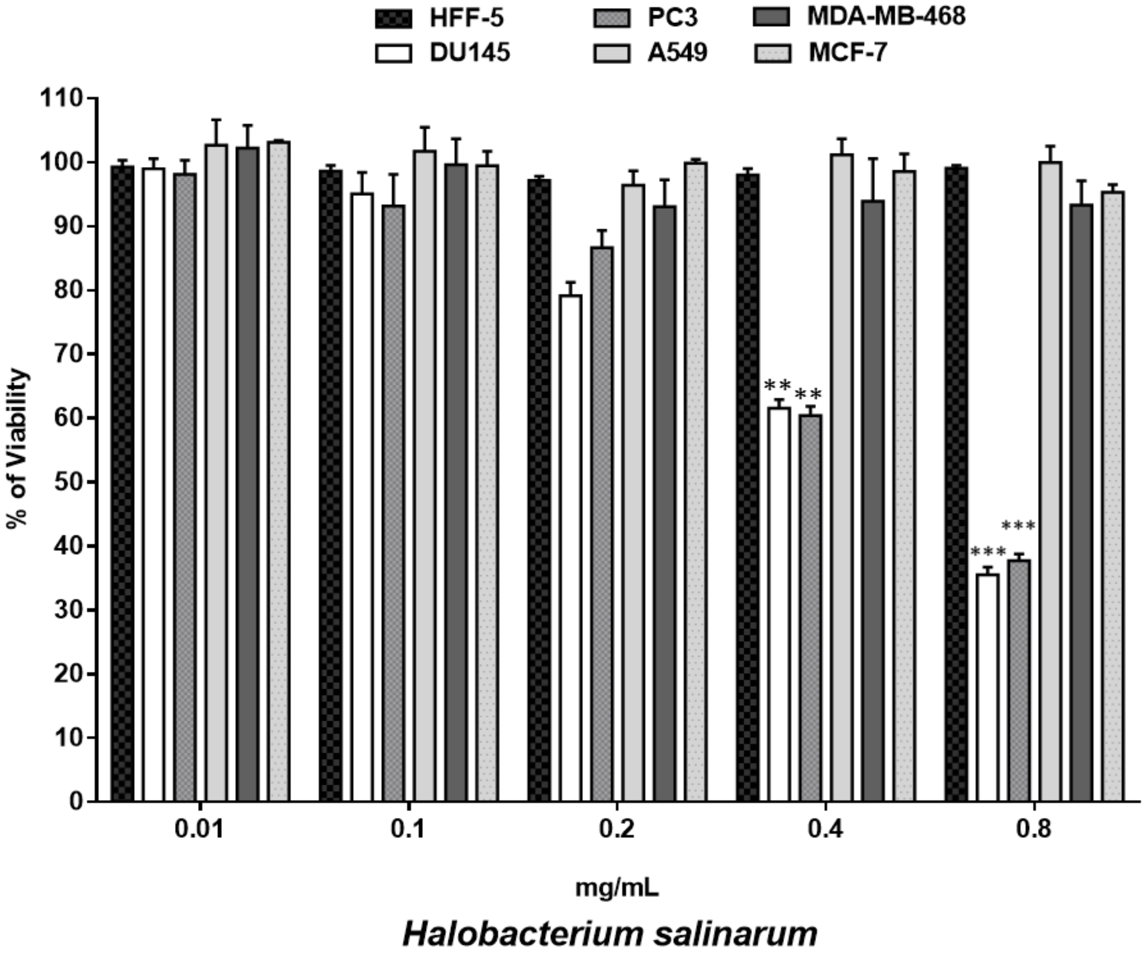

Figure S8. Screening of Supernatant Metabolites (SM) from *Halobacterium salinarum* on viability of prostate (PC3 and DU145), breast (MCF7 and MDA-MB-231), lung (A549) cancer cell lines and human foreskin fibroblast (HFF-5). The viability of cells measured 48 h post treatment with supernatant metabolites from *Halobacterium salinarum* by MTT. HFF-5 was used as normal control group. Bars indicated mean ± SD at least in five different biological replications. *p-values* showed significance of viability decrease. $^{**}p < 0.01$, $^{***}p < 0.001$
